# Articulation

**Published:** 1869-09

**Authors:** W. H. Thrift


### ARTICULATION.

BY W. H. THRIFT, D.D.S.

I have ventured to imagine, I may contribute a short article to the Register, that may not be entirely without interest to at least some of your readers.

The manner in which I obtain an articulation in full sets of teeth, is as follows: Over the plaster impressions mold temporary plates made of gutta percha, when cool, trim to suit case, using care that the plates do not interfere with the action of any muscles brought in contact with them, while in the mouth. Now select the teeth to be used in the case, and place the plates in the mouth, with incisor and molar sections temporarily fastened to them. Change the teeth if necessary in getting right articulation; take a strip of wax, warm it first over a spirit lamp, and fasten the two plates together by pressing the wax between incisor and molar sections, after giving the wax time to harden, remove the plates from the mouth together and plaster them on the articulator, not using plaster casts. After the teeth have been ground and arranged to suit, try them in the mouth, and if not right make the necessary changes before discharging patient.

I claim the following advantages over the old practice viz: time is saved, and it does away with the danger of marring impressions while grinding and arranging the teeth. It has been my experience that a more accurate articulation is obtained by this method than any other with which I am familiar.

---

A Madison, Wisconsin, dispatch to last evening's Chicago *Republican* says: "A sad accident occurred here this morning. One of Whitney's 'Vulcanizers'—a little steam boiler of copper, holding about a quart, and one-twentieth of an inch thick, used for vulcanizing rubber plates for false teeth—exploded in the office of Dr. Nelson Chittenden, instantly killing his office boy, Fred. Forsman, aged about eight years. The lower part of his face was torn away, his collar bone torn loose, and his lungs exposed. The boiler exploded with terrific force, sending two pieces of copper through a door connecting with another room."

[*Cin. Chronicle, Aug.* 4, 1869.

---

The Second Annual Meeting of the Missouri Valley Dental Society was held in the office of Dr. E. I. Woodbury, Council Bluffs, Iowa, July 13th and 14th.

E. I. Woodbury was elected President, C. Humas, of Nebraska City, Vice-President, and J. F. Sanborn, Tudor, Iowa, Secretary and Treasurer.

Dr. E. S. Williams, the retiring President, delivered an annual address on the Duties of the Profession.

The Society meets semi-annually on the 2d Tuesday and Wednesday in January and July.

